# Intratumoral Microbiota-Host Interactions Shape the Variability of Lung Adenocarcinoma and Lung Squamous Cell Carcinoma in Recurrence and Metastasis

**DOI:** 10.1128/spectrum.03738-22

**Published:** 2023-04-19

**Authors:** Xiangfeng Zhou, Lei Ji, Yanyu Ma, Geng Tian, Kebo Lv, Jialiang Yang

**Affiliations:** a Department of Mathematics, Ocean University of China, Qingdao, China; b Geneis Beijing Co., Ltd., Beijing, China; c Qingdao Geneis Institute of Big Data Mining and Precision Medicine, Qingdao, China; d Department of Mathematics, Zhejiang University of Science and Technology, Hangzhou, Zhejiang, China; e Chifeng Municipal Hospital, Chifeng, Inner Mongolia, China; f Academician Workstation, Changsha Medical University, Changsha, China; University of Arkansas for Medical Sciences

**Keywords:** non-small cell lung cancer, recurrence and metastasis, multi-omics, host-microbe interactions

## Abstract

Differences in tissue microbiota-host interaction between lung squamous cell carcinoma (LUSC) and lung adenocarcinoma (LUAD) about recurrence and metastasis have not been well studied. In this study, we performed bioinformatics analyses to identify the genes and tissue microbes significantly associated with recurrence or metastasis. All lung cancer patients were divided into the recurrence or metastasis (RM) group and the nonrecurrence and nonmetastasis (non-RM) group according to whether or not they had recurred or metastasized within 3 years after the initial surgery. Results showed that there were significant differences between LUAD and LUSC in gene expression and microbial abundance associated with recurrence and metastasis. Compared with non-RM, the bacterial community of RM had a lower richness in LUSC. In LUSC, host genes significantly correlated with tissue microbe, whereas host-tissue microbe interaction in LUAD was rare. Then, we established a novel multimodal machine learning model based on genes and microbes to predict the recurrence and metastasis risk of a LUSC patient, which achieves an area under the curve (AUC) of 0.81. In addition, the predicted risk score was significantly associated with the patient’s survival.

**IMPORTANCE** Our study elucidates significant differences in RM-associated host-microbe interactions between LUAD and LUSC. Besides, the microbes in tumor tissue could be used to predict the RM risk of LUSC, and the predicted risk score is associated with patients’ survival.

## INTRODUCTION

Lung cancer is the most common cancer in men worldwide, which mainly consists of two subtypes, small cell lung cancer (SCLC) and non-small cell lung cancer (NSCLC). NSCLC can be further divided into lung adenocarcinoma (LUAD), lung squamous cell carcinoma (LUSC), and large cell lung carcinoma (LCLC) (1). LUAD and LUSC combinedly account for around 70% of all lung cancer incidents (2). LUAD is a type of alveolar epithelial cell carcinoma derived from glandular differentiated or mucus-producing cancer cells, while LUSC is an epithelial basal cell carcinoma displaying keratinization and intercellular bridges (3). LUSC exhibits a higher progression rate than LUAD during tumor development, resulting in poorer outcomes for LUSC patients (4, 5). Therefore, it is essential to explore the differences between LUAD and LUSC to identify biomarkers guiding more personalized therapy.

In recent years, the comparisons between LUAD and LUSC have been widely studied at multiple molecular levels. For example, several studies have shown that there are significant mutational differences between LUSC and LUAD, with a significantly higher mutation rate in LUSC (6, 7). In addition, the differences between LUAD and LUSC in methylation patterns, copy number variation (CNV) mutation patterns, tumor immune microenvironment, and intratumor heterogeneity (ITH) have also been extensively studied (8–11). For instance, Zhang et al. found that ITH was higher in epidermal growth factor receptor (EGFR)-mutant LUAD than in LUSC (12). Despite the extensive effort being paid to this critical topic, there are still a few aspects being more or less ignored and worthy of more attention.

First of all, tissue microbial differences between LUAD and LUSC have not been deeply studied. Tissue microbiota has been suggested to influence the development, progression, metastasis, and treatment of multiple cancer types (13, 14). For example, Peters et al. (15) found that increased diversity and altered composition in normal lung tissue were associated with reduced disease-free survival (DFS) and recurrence-free survival (RFS). Bacterial abundance and diversity were significantly lower in tumor samples than in normal tissue (15). Studies have shown that the species Fusobacterium nucleatum, which was abundant in cancer tissues from patients who had relapsed from chemotherapy, could trigger cancer in multiple ways and was related to cancer cell invasion and metastasis (16, 17). Recently, Patnaik et al. found that the bacterial composition in the lower airways was associated with the recurrence of non-small cell lung cancer (NSCLC) after resection (18). Nonetheless, the potential relationship between microbial communities in cancer tissues and the recurrence or metastasis (RM) of LUAD and LUSC continues to be a knowledge gap.

Second, to our best knowledge, a comparison of tumor recurrence and metastasis between LUAD and LUSC has not been performed, especially at tissue microbial and host gene-tissue microbe levels. Recurrence and metastasis are the leading causes of lung cancer-related deaths, and tumor recurrence is the most common cause of surgical failure (3, 19, 20). Li et al. found that high expression of *CCL2* or *CCL4* was associated with poor prognosis in LUAD patients, while high *CCL2* or *CCL4* in LUSC was significantly associated with favorable progression-free survival (21). A research study showed that *TWF1* gene expression was significantly increased in both LUAD and LUSC and was significantly associated with the recurrence of LUAD, while no such association was found in LUSC (22). In addition, Jin et al. found that pulmonary commensal microbes drive the proliferation and activation of Vγ6Vδ1 T cells in lung cancer, while an increased abundance of γδ T17 cells was positively correlated with tumor invasion and metastasis in patients with LUAD (23, 24). Thus, comparing LUAD and LUSC in recurrence or metastasis at the tissue microbial and host gene-tissue microbial levels can help deepen our understanding of the mechanisms of recurrence or metastasis in these two cancer types.

In cancers, tissue microbes and host genes interact extensively, which might be crucial to cancer recurrence and metastasis. For instance, Dayama et al. found a significant negative correlation between the abundance of *Ruminococcaceae* in the gut and the *LCN2* gene, which is enriched for metastasis, tumor suppression, and other functions related to the gastrointestinal tract and colorectum in colorectal cancer (25). Recently, it has been shown that microbes such as *Acidovorax* exhibit higher abundance in tumors with *TP53* mutations, which have been proven to be significantly associated with the recurrence of many cancers such as endometrial carcinoma and oral cancer (26–28). However, the interaction between host gene expression and tissue microbes in the recurrence or metastasis of LUAD and LUSC has been rarely studied.

Besides, with the development of sequencing technology, there have been several studies using machine learning models combined with multi-omics data to predict recurrence or metastasis in LUSC patients. Shi et al. predicted recurrence or metastasis in LUSC patients by integrating information on mRNA, microRNA (miRNA), methylation, and copy number variation in LUSC patients in combination with the support vector machines (SVM) classifier (29). In addition, Yang et al. used SVM with decision tree models to predict tumor recurrence in LUSC patients by integrating clinical data and information on mutations and copy number variants in 15 genes in LUSC patients (10). Nevertheless, studies predicting recurrence or metastasis based on tissue microbiota with mRNA data and comparisons between different omics data are still lacking.

To resolve the above-mentioned issues, for LUAD, 123 paired transcriptome and tissue microbiota samples were collected, including 75 recurrence or metastasis (RM) samples and 48 nonrecurrence and nonmetastasis (non-RM) samples. For LUSC, 110 paired transcriptome samples and tissue microbiota samples were collected, of which 41 were RM samples and 69 were non-RM samples (30). The primary objectives of this study were (i) to identify the genes and tissue microbes significantly associated with recurrence or metastasis in LUAD and LUSC samples, respectively, (ii) to compare the differences between LUAD and LUSC in gene expression and tissue microbiota, and (iii) to identify novel biomarkers capable of predicting a lung cancer patient’s prognosis. This study helps us to expand our understanding of the mechanisms of recurrence or metastasis in NSCLC and contributes to targeted therapies in the clinic.

## RESULTS

### LUAD and LUSC revealed significant differences in gene expression and tumor tissue microbiota.

**FIG 1:**
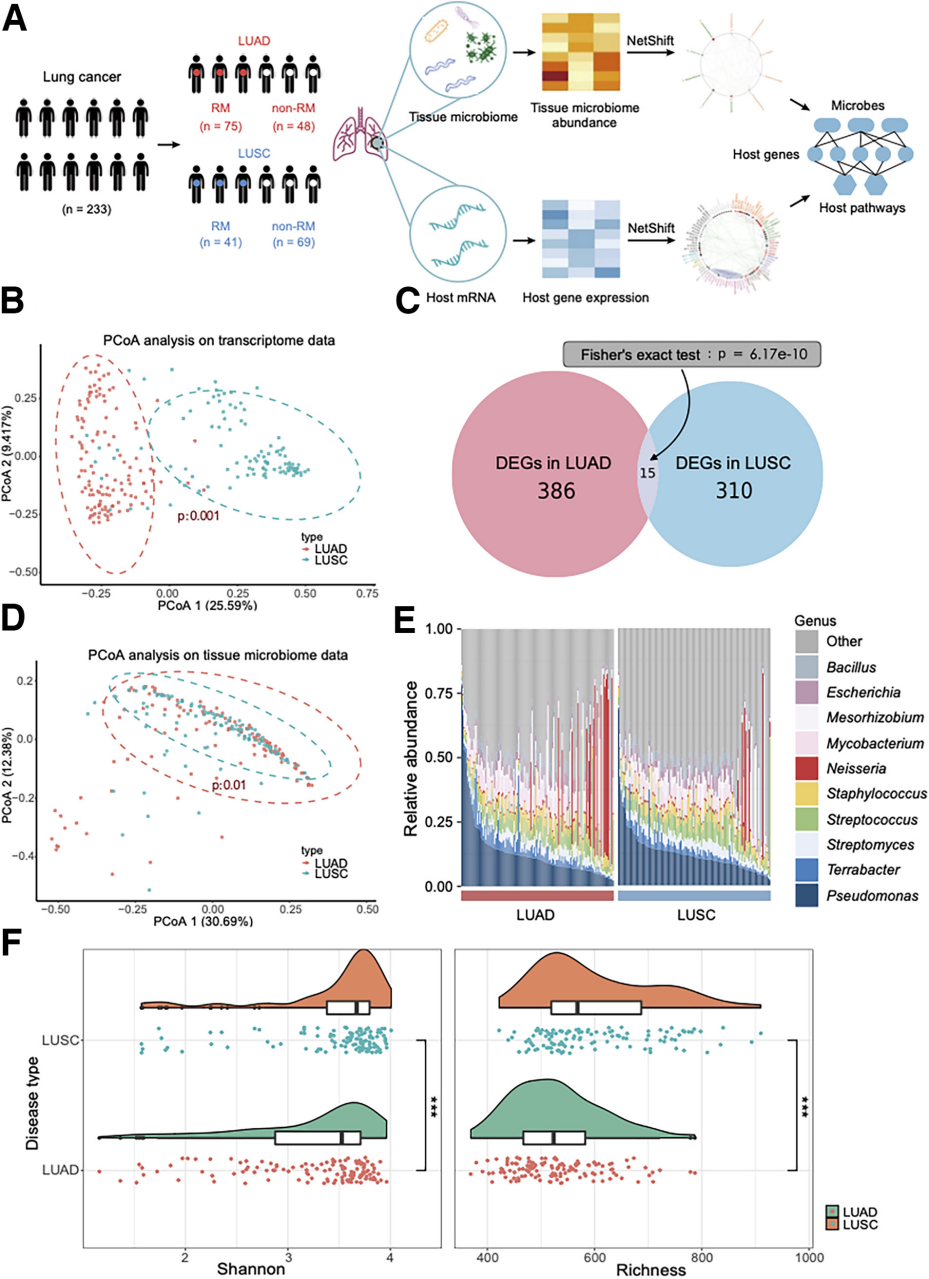
LUAD and LUSC differ significantly in gene expression and tissue microbiome. (A) General overview of study design: LUAD and LUSC samples were collected from TCGA, and paired transcriptome (RNA-seq) data and tissue microbiome abundance (16S rRNA) data were obtained for each sample. Paired transcriptome and tissue microbiome data were combined using correlation analysis to depict the association between tissue microbes and host genes and pathways in both diseases. (B) PCoA of transcriptome data. (C) Venn diagram showing the overlap of differentially expressed genes (DEGs) associated with tumor recurrence or metastasis in LUAD and LUSC patients (Fisher’s exact test, *P* value = 6.17e−10). DEGs were obtained by the DESeq2 method. (D) PCoA of tissue microbial data. (E) Stacked histogram to demonstrate the relative abundance of top 10 genus levels in tumor tissue of LUAD (left) versus LUSC (right) patients. (F) Microbial community diversity of LUAD versus LUSC.

The host transcriptome and tissue microbiota of LUAD and LUSC were significantly different for recurrence and metastasis. Principal-coordinate analysis (PCoA) was used in gene expression and tumor tissue microbiome data, respectively; we found that patients with LUSC and LUAD were distinguishable (the *P* value of PCoA is 0.001 in the gene expression data and 0.01 in microbiome data) (Fig. 1B and D). We analyzed the differentially expressed genes (DEGs) between RM and non-RM. Four hundred one DEGs were identified in LUAD mRNA expression, and 325 DEGs were identified in LUSC mRNA expression (Fig. 1C). Only 15 genes were significantly overlapping in the two sets of DEGs (Fisher’s exact test, *P* = 6.17e−10). Stacked bar charts were used to demonstrate the composition of microbes in cancerous tissue from types of patients at the genus level (Fig. 1E). The top 10 genera in relative abundance were similar in LUAD and LUSC, but microbial community diversity in LUSC was significantly higher than in LUAD (Fig. 1F). These results revealed that significant differences between LUAD and LUSC were in terms of both gene expression profiles and tissue microbiome profiles.

### RM and non-RM demonstrated significant differences in the microbiome profiles of LUSC.

**FIG 2:**
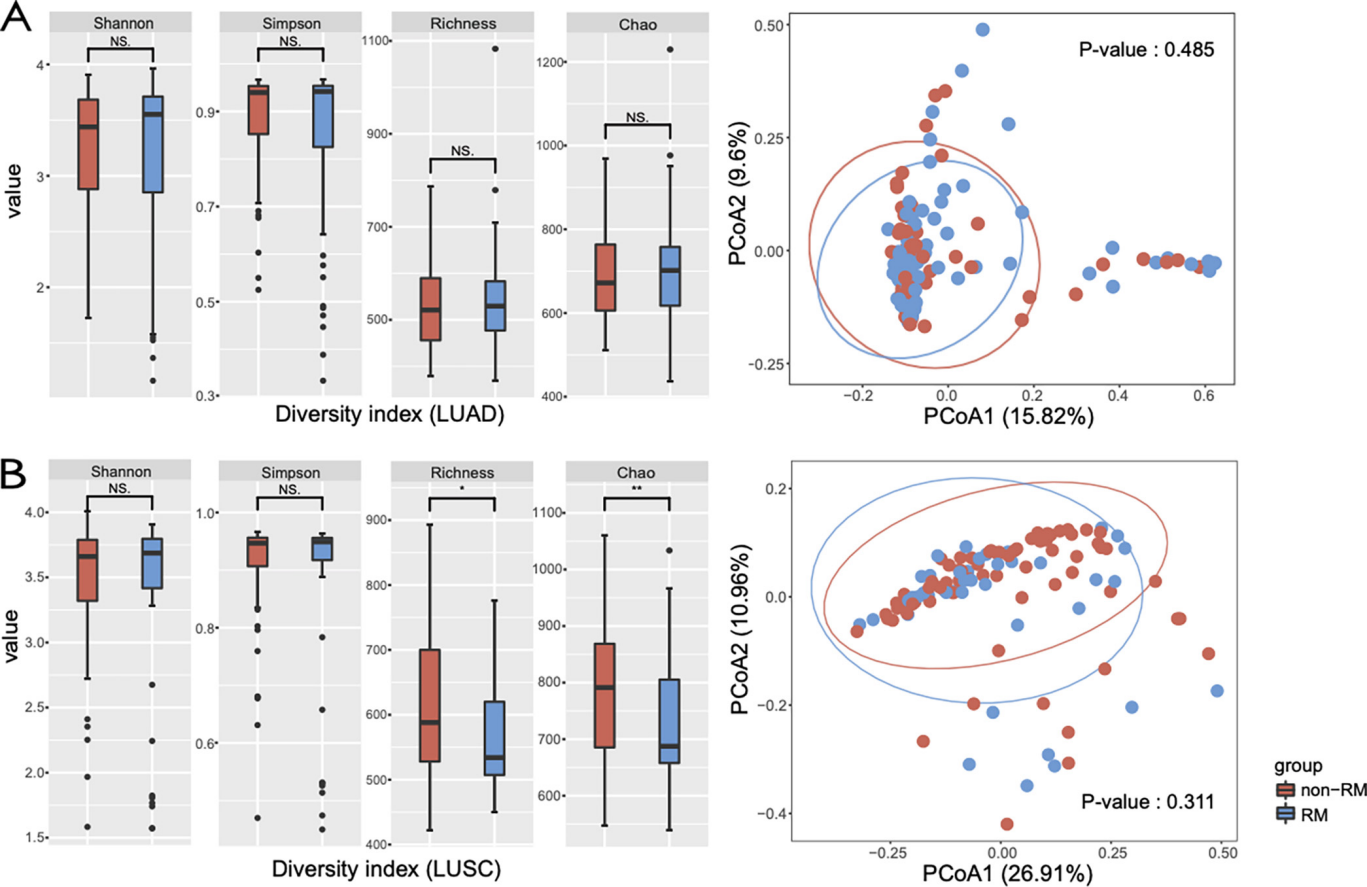
Microbial community diversity of LUAD and LUSC. (A) Microbial community diversity of LUAD. Comparison of alpha diversity of non-RM and RM based on different indexes (left); comparison of beta diversity of non-RM and RM with PCoA (right). The Wilcoxon test was used to measure the difference in microbial composition between the two groups based on genus level. (B) Microbial community diversity of LUSC. Richness and Chao indexes represent the richness of the microbial species; Simpson and Shannon indexes reflect the diversity of the microbial species.

Alpha diversity was calculated across LUAD and LUSC. In LUSC, we did not find a clear difference between RM and non-RM in the Shannon index and Simpson index. However, the richness index and Chao index of RM and non-RM revealed notable differences. Meanwhile, PCoA (*P* = 0.31) suggested no significant differences between the bacterial communities of RM and non-RM in LUSC (Fig. 2B). Conversely, there were no clear differences between RM and non-RM both in alpha diversity and in beta diversity in LUAD microbial communities (Fig. 2A). These results demonstrated an association between tumor recurrence or metastasis and the tissue microbiome diversity in patients with LUSC. The microbe diversity of cancer tissue was significantly lower in patients who developed tumor recurrence or metastasis than in those who did not.

### Integrating host gene expression and tissue microbiome abundance.

**FIG 3:**
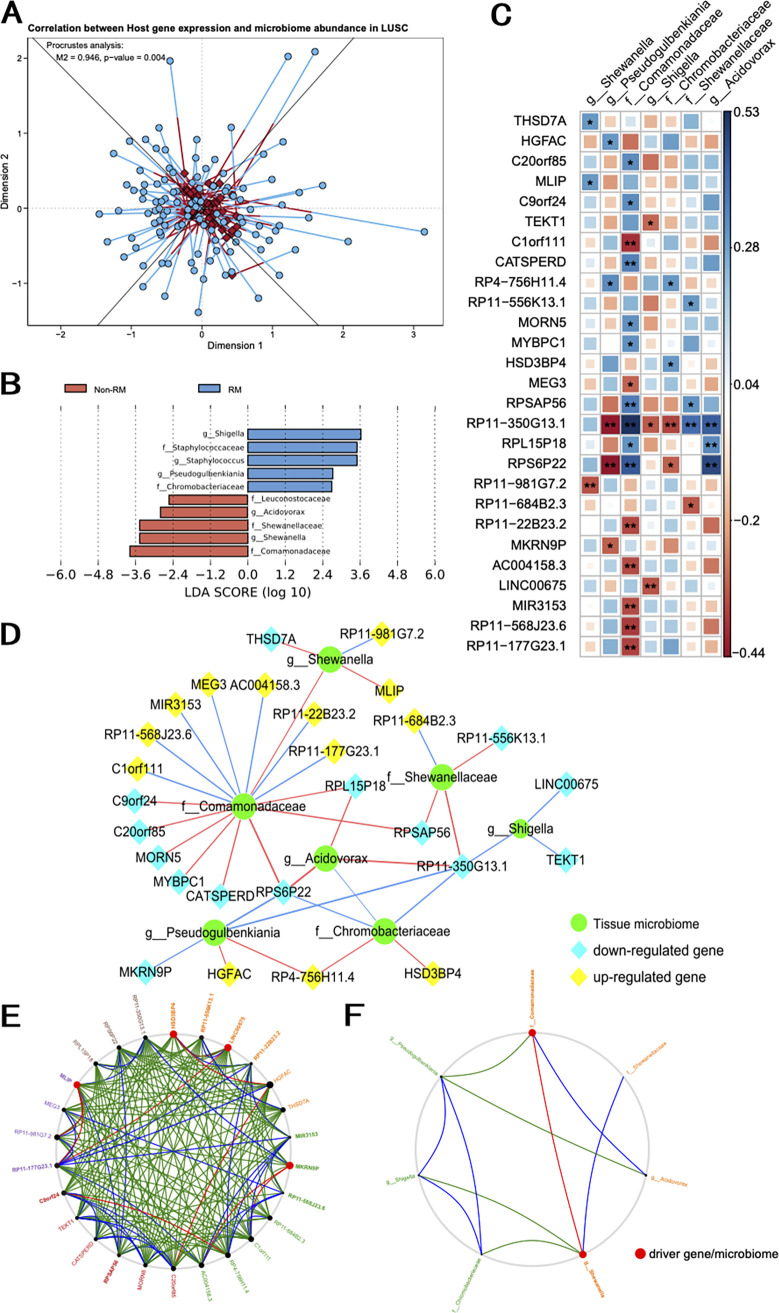
Interactions between host genes associated with tissue microbes in LUSC. (A) Procrustes analysis showing overall association between host gene expression and tissue microbiome composition in LUSC. Euclidean distance was used for host expression data (diamonds), and Bray-Curtis distance was used for tissue microbiome data (circles). (B) LDA scores for the bacterial taxa differentially abundant between RM and non-RM in LUSC (LDA score of >2.5). Blue bars indicate microbes significantly enriched in the RM group, and red bars indicate microbes significantly enriched in the non-RM group. (C) Correlation plot portraying gene-microbe correlations. Color and size of squares indicate the scale of the correlation, and the asterisks indicate significance of correlation (** denotes *q* value of <0.05, and * denotes *q* value of <0.1). (D) Visualization of significant gene-microbe correlations using the network. In the network, blue edges denote negative correlations and red edges denote positive correlations. The thickness of the line represents the strength of the correlation. (E and F) Using the network shift (NetShift) method to capture changes between the two cooccurrence networks that correspond to the RM and non-RM patients. Black nodes indicate nodes that are present in both the Case and Control subnets. The size of a node is proportional to its corresponding neighbor shift (NESH) score, and we define a node with a NESH score greater than 1.6 as a drive node. The color of the edges represents the interworking status of the nodes, with red indicating interworking in the RM subnetwork only, green indicating interworking in the non-RM subnetwork only, and blue indicating a tight relationship in both subnetworks. (E) NetShift analysis of host genes in association networks. (F) NetShift analysis of tissue microbes in association networks.

Procrustes analysis was performed to find an overall association between host gene expression and tissue microbial composition in LUAD and LUSC. The results of our analysis showed a remarkable connection between host gene expression and tissue microbial composition across subjects in LUSC (*P* = 0.004) (Fig. 3A). The Mantel test was conducted to verify this result. However, the result of the Procrustes analysis was not significant in LUAD (*P* = 0.09; see Fig. S1A in the supplemental material). The lack of a significant overall connection between host gene expression and tissue microbiota in LUAD might indicate that, instead of an overall correspondence between the two, it is probable that only a subset of tissue microbes is connected with a subset of host genes in LUAD.

### Bacteria were significantly associated with DEGs in LUSC.

In LUSC, *Shigella*, *Staphylococcaceae*, Staphylococcus, *Pseudogulbenkiania*, and *Chromobacteriaceae* were enriched in RM, while *Leuconostocaceae*, *Acidovorax*, *Shewanellaceae*, *Shewanella*, and *Comamonadaceae* were enriched in the non-RM group (Fig. 3B). Among them, the relative abundance of *Comamonadaceae* was more abundant in non-RM. However, in LUAD, there were only *Comamonadaceae* and *Acidovorax* being significantly enriched in non-RM (Fig. S1A). DEGs in LUSC patients potentially interacted with differential bacteria in tissues (Fig. 3C). Using Spearman correlations, we found 38 significant host gene-tissue microbiota correlations in LUSC (false-discovery rate [FDR] [*q* value] of <0.1). We visualized all the correlations between taxon abundance and host gene expression in Fig. 3D. There were 27 genes and seven microbes in the network (here referring to novel recurrence or metastasis-related signature [34RMSig]). In particular, we found that in the gene-microbe network, microbial nodes have more edges on average than do genes, where *Comamonadaceae* formed distinct hubs in the network. The family *Comamonadaceae* now includes the genera *Comamonas*, *Delftia*, and *Acidovorax*. Previous studies have shown that the relative abundance of *Acidovorax* was higher in smokers, and squamous cell carcinoma (SCC) tumors from smokers have an even greater relative abundance of these bacteria (26). To understand the role of important genes and species in the host-microbe correlation network in LUSC, we used the network shift (NetShift) method to identify important species and genes in the network. Impressively, the *Comamonadaceae* family and *Shewanella* genus displayed high neighbor shift (NESH) scores as the “driver” microbes in our network. Equally, we found HSD3BP4, LINC00675, MKRN9P, and MLIP as the “driver” genes (Fig. 3E and F).

### Tissue microbes were associated with pathways.

**FIG 4:**
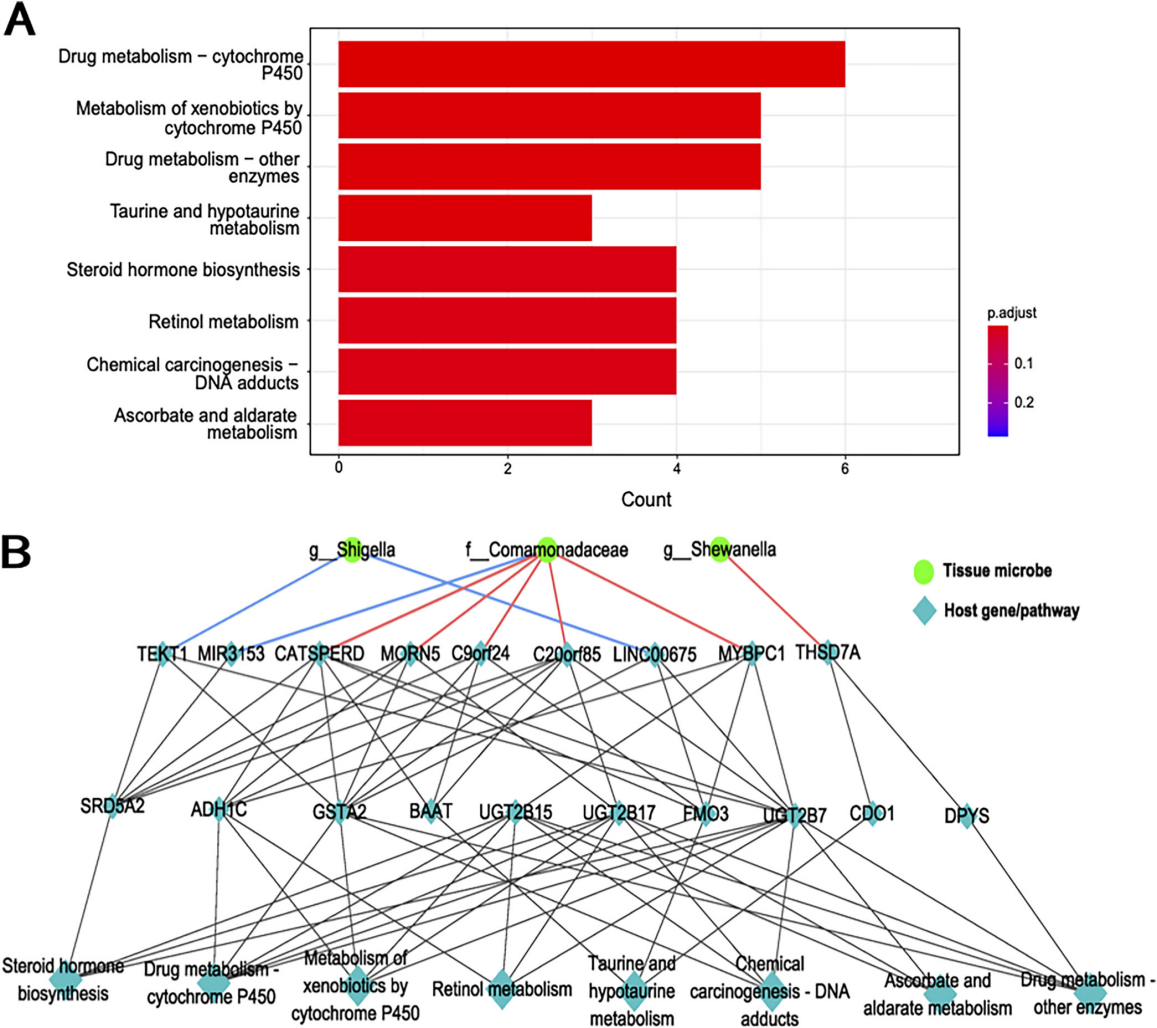
Tissue microbes are associated with host genes and pathways in LUSC. (A) Host pathways enriched among DEGs that are significantly correlated with genes in host gene-tissue microbiome associations in LUSC (FDR of <0.05). (B) Network showing tissue microbes correlated with host pathway in LUSC.

We applied enrichment analysis to characterize the biological functions represented by the host genes which associate with specific tissue microbes. Within the DEGs in LUSC, we found 175 genes significantly correlated with genes in 34RMSig (Spearman correlation, *q* value of <0.05), and then we identified eight host pathways that associated with tissue microbes utilizing these selected genes (Fig. 4A; Fisher’s exact test, Benjamini-Hochberg FDR of <0.05). The host pathways we identified were known to participate in metabolic processes, such as cytochrome P450 (CYP), which was implicated in the development and progression of tumors and the activation of anticancer prodrugs and their metabolic clearance (31–33).

### 34RMSig data were precise predictors of recurrence and metastasis in LUSC.

**FIG 5:**
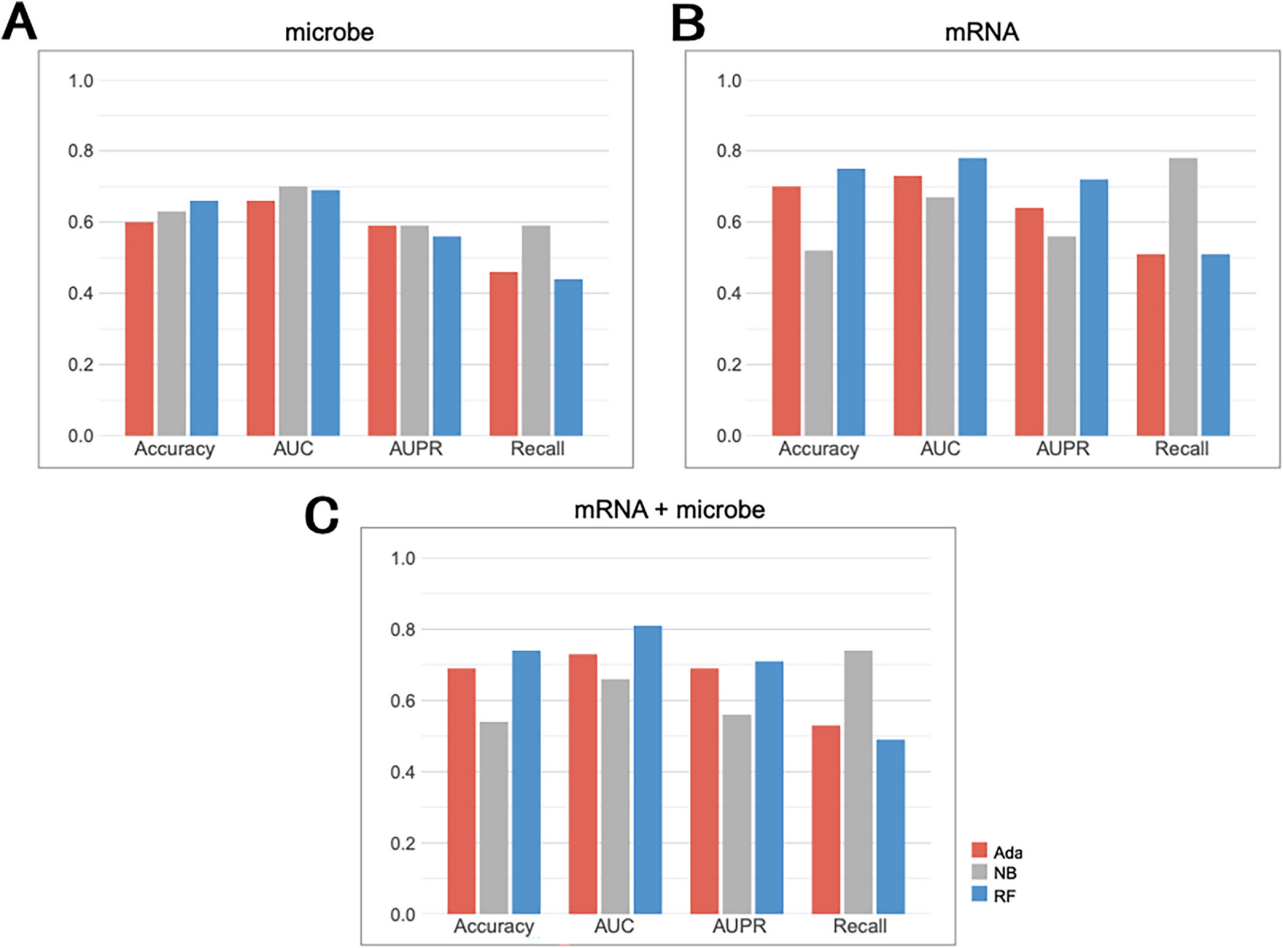
Comparison of recurrence or metastasis predictive ability for multi-omics data. Multi-indicator evaluation of the predictive ability of three classifiers for microbial data (A), mRNA data (B), and combined microbial and mRNA data (C) for tumor recurrence or metastasis in LUSC patients. Ada, Adaboost model; NB, naive Bayes model; RF, Random Forest model.

We evaluated the performance of 34RMSig data in predicting cancer recurrence or metastasis in patients with LUSC. We selected microbes and mRNA from 34RMSig for modeling separately. The performance of each data set was measured by the average scoring indicator of 5-fold cross-validation (5-fold-cv) (Fig. 5A and B). Results showed that mRNA performed better than microbiome. The Random Forest (RF) classifier performed better than other machine learning models on mRNA data, with an area under the curve (AUC) of 0.78 on a 5-fold-cv (Fig. 5A). The best AUC of the microbiome was 0.7 (Fig. 5B). Additionally, when combining the mRNA and microbiome, the RF model achieved an AUC value of 0.81, which was higher than the value obtained with mRNA or microbiome alone (Fig. 5C). Results indicated that both gene expression and microbiome abundance information can be used to predict recurrence or metastasis in patients with squamous lung cancer, and mRNA should be prioritized to do such work. Combining the two omics data sets can yield better classification results.

Furthermore, we calculated the feature importance of each gene or microbiome in the machine learning classification model, as shown in Fig. S2A and C. The importance of each feature is represented by the feature information. As a statistical indicator describing the ability of an attribute to distinguish data samples, the information gain can describe the ability of the feature to distinguish sample data. We can see that among the feature importance values of the gene set used to construct the classification model, the feature importance of the gene obtained by the NetShift method ranks in the top 10. Similarly, among the feature importance values of the gene set used to construct the classification model, the feature importance of *Shewanella* and *Comamonadaceae* accounts for half of the total microbial feature importance. In addition, we removed the features obtained by NetShift from the classification model and used the same model to build the classification model. We found that the AUC of the prediction model significantly decreased, as shown in Fig. S2B and D. (The maximum AUC value of the model on the gene set is 0.59, and the maximum AUC value of the model on the microbial set is 0.59).

Therefore, we believe that the features obtained by the NetShift method have a good performance in predicting the recurrence and metastasis of LUSC patients.

### 34RMSig data were strongly significantly associated with patient survival time.

**FIG 6:**
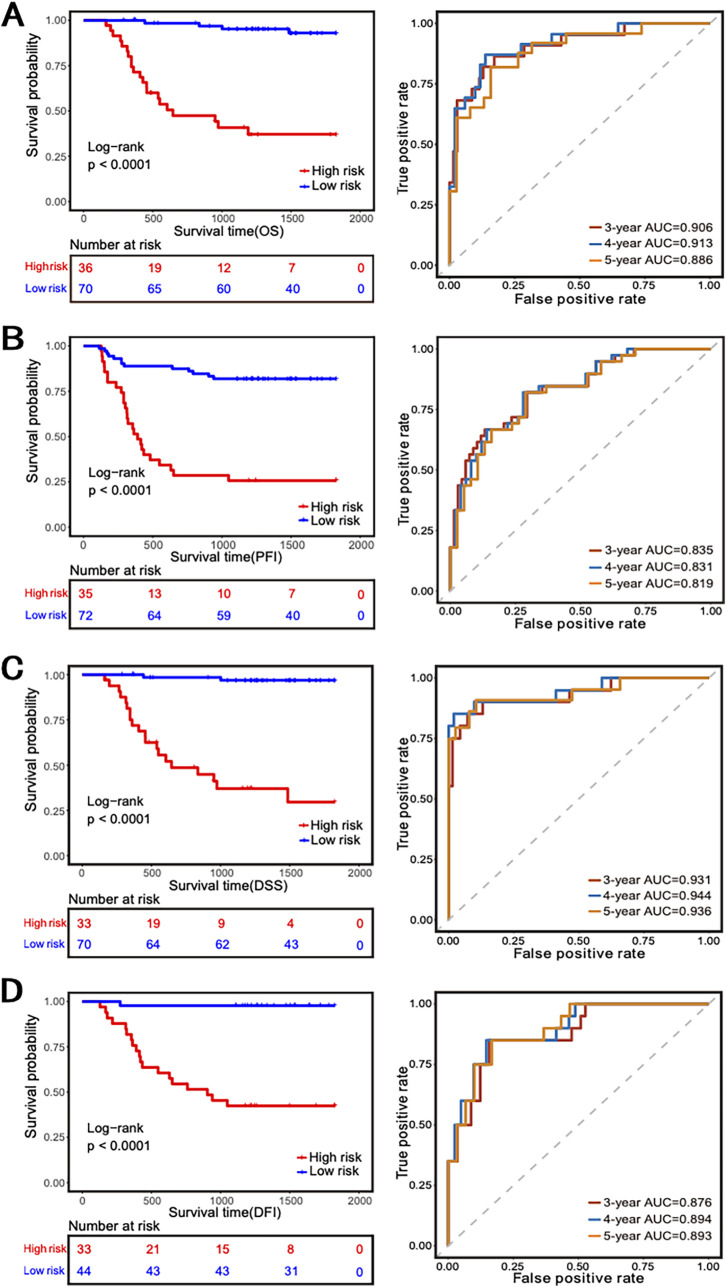
Performance evaluation of 34RMSig. (A to D) (Left) Kaplan-Meier survival curves of patients in the high-risk and low-risk groups among OS (overall survival), PFI (progression-free interval), DSS (disease-specific survival), and DFI (disease-free interval). (A to D) (Right) Receiver operating characteristic curve for the performance of the 34RMSig for 3-, 4-, and 5-year survival.

To investigate the potential relationship between 34RMSig and patients’ survival time, 34RMSig was transformed into a risk-scoring model. The optimal risk cutoff value stratified patients into the high-risk group and low-risk group with different survival times (Fig. 6). Among the four sets of survival data, patients in the high-risk group had conspicuously poorer survival than those in the low-risk group (log rank test *P* value of <0.001).

The time-dependent receiver operating characteristic (ROC) analysis showed the performance of 34RMSig for predicting 3-, 4-, and 5-year overall survival, progression-free interval, disease-specific survival, and disease-free interval. Compared to 3- and 5-year values, 34RMSig achieved higher AUC scores in predicting 4-year survival status in overall survival, disease-specific survival, and disease-free interval, with AUC values of 0.913, 0.944, and 0.894, respectively.

## DISCUSSION

Currently, the treatment methods for LUAD and LUSC mainly include chemical therapy, radiation treatment, and surgical treatment. However, a high proportion (30% to 60%) of patients will develop a local and/or distant recurrence after surgery, resulting in a long-term survival rate of less than 50% for patients after surgery (34–37). Though there are many studies of NSCLC recurrence or metastasis, differences between LUAD and LUSC in recurrence or metastases are rarely explored. Our study fills this vacancy and finds biomarkers at the tissue microbiota level. Based on transcriptome sequencing (RNA-seq) and microbiome abundance data of LUAD and LUSC patients, we analyzed the differences in host gene expression and tissue microbes between the two groups of patients. DEGs associated with tumor recurrence or metastasis were significantly different in the two patient groups. In LUSC patients, the richness of bacterial communities of non-RM was higher than that of RM, and no significant difference was found between non-RM and RM in patients with LUAD. Again, we found there were no significant differences between the two groups in beta diversity. Then, we creatively identified a host-microbe association in LUSC patients by integrating data from these two omics data sets, and there was no significant correlation in LUAD. Further, we identified the driver genes and microbes in the host-microbe interaction network and observed significant differences in several driver nodes such as LINC00675 and *Comamonadaceae*.

We established classification models to predict patients’ recurrence or metastasis in LUSC and compared the predictive performance of mRNA and microbiome data in 34RMSig. Results showed that mRNA has a better predictive performance than tissue microbiome, and the prediction of the model was further enhanced when combining two omics data sets, with an AUC value of 0.81 on 5-fold-cv, which suggested that host gene expression and tissue microbiome abundance information were complementary in predicting recurrence or metastasis in LUSC. To further investigate the clinical application of these correlated genes and microbes, we used the 34RMSig to stratify LUSC patients into two risk groups, and the 34RMSig revealed a significant association with survival and could distinguish between LUSC patients with good and poor outcomes.

We also identified a set of pathways that were associated with specific tissue microbiome composition in LUSC. These pathways were mostly related to various metabolic and metabolism-related enzymes, and some of them have been previously implicated in lung cancer: for example, drug metabolism-cytochrome P450, metabolism of xenobiotics by cytochrome P450, and steroid hormone biosynthesis. Previous studies found that CYP (cytochrome P450) metabolizes carcinogens to their inactive derivatives but sometimes converts chemicals to carcinogens. Additionally, CYP is also involved in the activation and/or inactivation of anticancer agents, which indicates that the expression of CYP in lung cancer and paraneoplastic tissues may be an important factor in the efficacy of anticancer drugs. CYP24, which is frequently expressed in lung cancer tissues, can convert 25-dihydroxyvitamin D3 and 1alpha into inactive 24-hydroxylated derivatives, and 1alpha and 25-dihydroxyvitamin D3 inhibit the proliferation of cancerous tissues (31, 32, 38). Besides, steroid hormones include glucocorticoids, salt corticoids, progestins, androgens, and estrogens (39). There is growing evidence that the lung contains receptors for estrogen and progesterone and that these hormones have a role in lung development, lung inflammation, and lung cancer (40). It was shown that cytoplasmic estrogen receptor (ER)-α, cytoplasmic ER-β, and total progesterone receptor (PR) expression were higher in squamous cell carcinoma than in adenocarcinoma (AD), while HER2 expression was higher in adenocarcinoma than in squamous cell carcinoma (41). Furthermore, steroid hormones are associated with poor prognosis in lung cancer patients, and one study demonstrated that ERβ-1 was associated with a poorer prognosis in women with stage I lung cancer. Nuclear ERβ-1 was also associated with poor survival in patients with metastatic lung cancer but not in patients with early-stage lung cancer (40).

Over the past few decades, the study of microbes has provided many insights into research on lung cancer. The importance of the interaction between the host and microbiome in the pathogenesis of lung cancer has become increasingly apparent (42). Studying the interaction between host genes and tissue microbes in the recurrence or metastasis of lung cancer can fill out the understanding of cancer occurrence, development, and diagnosis.

The genus *Comamonadaceae* is of interest in our study. Previous studies found that in lung cancer, the relative abundance of the *Comamonadaceae* in paracancerous tissue was significantly higher than that in cancerous tissue areas, and *Comamonadaceae* had the highest linear discriminant analysis (LDA) score among several taxa associated with tumor status (43). The family *Comamonadaceae* included the genera *Comamonas* and *Acidovorax*, which were differentially abundant in squamous cell carcinoma (SCC) versus adenocarcinoma (AD) in non-small cell lung cancer. Of these, *Acidovorax* is well differentiated between smoker and nonsmoker populations, with higher levels of *Acidovorax* in the lungs of smokers than of nonsmokers. In addition, *Acidovorax* and *Comamonas* were also more abundant in tumors carrying TP53 mutations, the most common somatic mutation in lung squamous cell carcinoma and strongly associated with prognostic survival of lung squamous carcinoma patients, suggesting that *Acidovorax* and *Comamonas* are specific for lung SCC with TP53 mutations (26, 44, 45). Although there is no direct evidence that these microorganisms promote recurrence or metastasis of lung SCC or induce TP53 mutations, they are significantly associated with factors that contribute to recurrence or metastasis and poor prognosis in patients with squamous lung cancer, such as smoking and TP53 mutations. Therefore, we believe that these tissue microbes can be used as markers to identify recurrence or metastasis in LUSC.

In host-microbe associations, maternally expressed gene 3 (MEG3) was significantly negatively correlated with *Comamonadaceae*. MEG3, located in the imprinted region of chromosome 14 DLK1-MEG3, is a long noncoding RNA (lncRNA) that is expressed in many normal tissues. Recently, several studies have shown that MEG3 expression level is reduced in lung cancer, hepatocellular carcinoma, and gastric cancer (46–48). In NSCLC, MEG3 regulates cell proliferation and apoptosis through activation of TP53, and patients presenting low levels of MEG3 expression had a comparatively poor prognosis. Besides, compared with normal tissue, the expression of MEG3 is reduced in non-small cell lung cancer (NSCLC) tumor tissues (49).

We speculate that the association between MEG3 and *Comamonadaceae* in lung squamous carcinoma patients might be able to be described by TP53: in NSCLC patients with deletion of MEG3 expression and relative increase in *Comamonadaceae* due to mutations in the TP53 locus, there is a negative correlation between MEG3 expression and *Comamonadaceae* abundance, in agreement with our findings in host gene-tissue microbial association analysis in Fig. 3D.

LINC00675 was significantly negative with the genus *Shigella*. LINC00675, also known as TMEM238L, is a long intergenic noncoding RNA found in the 17p13.1-p12 region of the human chromosome. Previous studies have shown an association between abnormal LINC00675 expression and poor prognosis in a variety of cancers. LINC00675 is involved in the proliferation and migration of cancer cells by promoting the activity of the Wnt/β-catenin signaling pathway to activate downstream, targeting c-*myc* and cyclin D1 (50, 51). In gastric cancer, LINC00675 enhanced the phosphorylation of vimentin on Ser83, while in colorectal cancer, LINC00675 inhibited the development of colorectal cancer by sponging miR-942 and regulating Wnt/β-catenin signaling (46, 52). miR9-942-5p is a microRNA (miRNA) that has now been shown to regulate cell proliferation and apoptosis in esophageal squamous cell carcinoma, human liver fibrosis, and non-small cell carcinoma (53, 54). In recent years, it has been shown that miR-942 is highly expressed in NSCLC, and serum miR-942 expression levels are significantly upregulated in NSCLC and correlated with unfavorable clinical variables and low survival in NSCLC (55). Furthermore, in a study of the NSCLC tumor microenvironment, the M2 macrophage-derived exosome miR-942 was found to promote the migration and invasion of LUAD cells and promote angiogenesis (56). Overexpression of miR-513b significantly inhibits proliferation, invasion, and migration and promotes apoptosis in non-small cell lung cancer (NSCLC) cells (57). LINC00675 has been previously found to be aberrantly expressed with miR-513b-5p in several types of cancer and regulates the malignant phenotype of cancer cells (58). Therefore, we hypothesized that LINC00675 might be able to influence tumor recurrence or metastasis in NSCLC by regulating miR-942 and miR-513b.

THSD7A promotes angiogenesis, which is closely associated with the tumor microenvironment, growth, and metastasis in NSCLC, and tumor-associated angiogenesis promotes tumor progression and metastasis (59–61). *Shewanella* bacteria are Gram negative and commonly found in the marine environment, ice-cold fish, and protein foods, and their role in the development of cancer is poorly studied. Recently, it has been shown that *Shewanella* and *Halomonas* are symbiotic in lung tissue and that microbes belonging to the genera *Halomonas* and *Shewanella* predominate in the lung tissue of patients with lung cancer (62).

The relative abundance of *Shewanella* and *Halomonas* was significantly higher in rectal and distal patients; however, in lung cancer patients, the relative abundance of *Halomonas* was significantly lower in lung tumor tissue than in normal tissue near the tumor, contradicting the results we obtained previously (63, 64). We hypothesize that in addition to the abundance of *Halomonas*, *Shewanella* abundance in lung tumor tissue of lung cancer patients is influenced by other factors, such as dysregulated immune responses (65). Therefore, the relationship between THSD7A and *Shewanella* needs to be further explored.

Our study has some limitations. First, our data are limited, limiting the generalizability and applicability of our model. Some factors that may influence microbial abundance or host gene expressions such as patient medication history and dietary habits were not available to us. Second, the tissue microbiome data in our study had only genus-level taxonomic resolution. The absence of species-level bacterial data reduces the depth to investigate differences in host-microbial interactions between LUAD and LUSC for recurrence and metastasis. In the future, the addition of species-level bacterial abundance data can further validate our conclusion, that is, to test the reliability of the genus-level bacteria excavated in this work. Finally, given that studying causality in humans is challenging, future studies using *in vivo* or *in vitro* models may be used to investigate the causal relationship and direction of action between specific host genes and microbes.

In conclusion, we performed a comprehensive analysis and found significant differences in gene expression and tissue microbiome between LUAD and LUSC for recurrence or metastasis. In LUSC, the analysis was capable of finding an interaction between host genes and tissue microbes, whereas in LUAD there was no such relationship. Besides, our results suggest that microbiome data can be used as a complementary explanatory variable in the prediction of tumor recurrence and metastasis in LUSC patients, improving the predictive power of the model. In addition, the genes and microbes in the association network can provide a good picture of the survival status of LUSC patients. This study can expand our understanding of the differences between LUAD and LUSC and provide new ideas for the study of tumor recurrence or metastasis in patients with LUSC.

## MATERIALS AND METHODS

### Sample collecting and preprocessing.

Research data were collected from the TCGA database. Paired transcriptome profiles and tissue microbiome data of LUAD and LUSC patients were obtained from previous studies (LUAD samples, 123 cases; LUSC samples, 110 cases) (30). Then, we used clinical information to determine tumor recurrence or metastasis, and using 3 years as the boundary, 75 of the LUAD patients had cancer recurrence or metastasis, while the corresponding number of LUSC patients was 41. We noted patients who suffered tumor recurrence or metastasis as RM and marked the annotations with patients who had no tumor recurrence and metastasis as non-RM.

### Differences in transcriptome and microbiome between LUAD and LUSC.

The R package ‘vegan’ (66) was used to identify the differences in transcriptome and tissue microbiome data between LUAD and LUSC. Differences in gene expressions and microbial communities were analyzed using principal-coordinate analysis (PCoA).

### Selection of DEGS.

We used the R package “DESeq2” (67) to identify the differentially expressed genes (DEGs) of mRNA. We used a *P* value of <0.05 and a log_2_ fold change of >1 as thresholds for upregulated genes and a *P* value of <0.05 and a log_2_ fold change of <1 for downregulated genes. Finally, expression levels with significant differences related to recurrence or metastasis were obtained according to a |log_2_(fold change)| of ≥1 and an adjusted *P* value of <0.05.

### Procrustes analysis and Mantel test.

To assess the overall correspondence between host gene and tissue microbe in LUAD and LUSC, we performed Procrustes analysis in R using the ‘vegan’ package. For each disease cohort, we used Euclidean distance for host gene expression data and Bray-Curtis dissimilarity for tissue microbiome data. The significance of the rotation agreement was obtained using the ‘protest ()’ function with 999 permutations. We also applied a Mantel test to verify the overall correlation between dissimilarity matrices of host gene expression and tumor tissue microbiome abundance in each cohort.

### Microbial diversity analysis.

Alpha diversity was used to analyze the diversity of the microbial community based on the operational taxonomic unit (OTU) counts in our samples. Additionally, we used PCoA to analyze differences in microbial communities. The LDA effect size (LEfSe) uses the Wilcoxon rank sum test and the nonparametric Kruskal-Wallis rank sum test method to analyze the differences between groups at different levels (68). An LDA score of >2.5 and a *P* value of <0.05 were considered to be statistically significant.

### Integrated analysis of interactions between significant host gene and microbiome in lung cancer recurrence or metastasis.

Correlation analyses were performed between differential tissue microbiomes obtained by LEfSe and DEGs. We computed the Spearman rank correlation coefficients and the corresponding *P* values using the ‘*cor.test ()’* function with a two-sided alternative hypothesis. *P* values were corrected for multiple comparisons using the ‘qvalue’ package in R. Remarkable gene-taxon correlations were demonstrated using ‘corrplots’ in R, where the direction of the correlation is represented by the color and thickness of the line indicating the strength of the correlation. What is more, the asterisk was used to mark a significant gene-taxon correlation. Finally, using Cytoscape v3.9.1 (69), we obtained the network for demonstrating significant gene-microbe correlations (*q* value of <0.1).

### Machine learning classification model and evaluation metrics.

We chose Random Forest (RF), Gaussian naive Bayes (NB), and Adaboost (Ada) as classification models for predicting RM status in LUSC patients.

NB classifier is based on classical mathematical theory and has a high practicality. The principle of the plain Bayesian approach to determine the class to which a sample belongs is by calculating the probability that the sample belongs to a certain class. Given data *x* = (*x*_1_, *x*_2_, …, *x*_d_), whose corresponding set of class labels is *C* = {*C*_1_, *C*_2_, …, *C*_k_}, and where *P*(*C_k_*) denotes the prior probability of *C_k_*(*K* = 1, 2, …, *K*), the conditional probability of *x* conditional on *C_k_* can be expressed as *P*(*C_k_*|*x*), and according to Bayes’ theorem, the following equation can be obtained:

$$P(C_{k}\;\;|\;\;x)=\frac{P(C_{k}\;\;|\;\;x)P(C_{k})}{P(x)}$$

If the individual feature attributes satisfy mutual conditional independence, the above equation can be expressed in the following form:

$$P(C_{k}\;\;|\;\;x)=\frac{P(C_{k})}{P(x)}\overset{m}{\underset{i=1}{\prod }}P(x_{i}\;\;|\;\;c)$$

Based on the above calculation, the plain Bayesian finds the maximum *P*(*C_k_*|*x*) for each item to be classified, which will be used as the classification prediction result. The NB model has the advantages of simplicity, speed, and robustness in training and testing large amounts of data and thus is widely used in the analysis of bioinformatics.

The Ada model algorithm starts with the same settings for the training data set and base classifiers and then changes the weight distribution while training to obtain different classifiers, resulting in a combined classifier with improved classification performance. The expression of the Ada model *G*(*x*) is as follows:

$$G(x)=\text{sign}(f(x))=\text{sign}(\overset{M}{\underset{i=1}{\sum }}\alpha_{m}G_{m}(x))$$

*G_m_*(*x*) denotes the *m*-th base classifier model in the Ada model, and α*_m_* is the weight of model *G_m_*(*x*) in *G*(*x*), which measures the importance of the classification result of *G_m_*(*x*) in the final classification result *G_m_*(*x*). The weights of the base classifier α*_m_* were updated by iterative operations.

Similar to the principle of the Ada model, the RF model trains multiple decision tree models and eventually determines the class to which the sample belongs by considering the classification results of all decision tree models together. Ada and RF models have excellent properties such as good classification, robustness, and low influence by the imbalance of sample categories. The depth of the Ada and RF models and the number of base classifiers (or decision trees) are important parameters that affect how well the models classify.

We choose to use the 5-fold cross-validation method to evaluate the average prediction performance of the model. The 5-fold cross-validation method involves randomly dividing the data set into five parts with equal sample sizes, using four parts at a time as the training set to train the model, and using the data from the remaining part as the validation set, cycling five times so that each part is used once as the validation set of the model. Fivefold cross-validation maximizes the use of the data set and reduces the overfitting of the training model. One hundred ten LUSC patients were used as the data set for cross-validation, and 27 DEGs and the *Shewanella* genus, *Pseudogulbenkiania* genus, *Acidovorax* genus, *Shigella* genus, *Comamonadaceae* family, *Shewanellaceae* family, and *Chromobacteriaceae* family were used as predictors. The results of the five cross-validations were averaged as the final results of the model. The specific tuning parameters are shown below.

As for the RF and Ada algorithms, we adjusted the tree number and the maximum depth of the tree. The evaluation metrics of the model include the area under the curve (AUC), accuracy (ACC), the area under the accuracy-recall curve (AUPR), and recall, which are used to evaluate the performance of the machine learning method in binary classification problems. AUC is the area covered under the ROC curve, which is used to measure the comprehensive classification performance of the model, ACC is the proportion of samples correctly predicted by the model to the total sample, and recall is the proportion of RM patients correctly predicted by the model to all RM patients. AUPR is an evaluation metric used to measure the comprehensive classification performance of the sample in the case of unbalanced sample categories.

### Survival analysis.

We selected genetic and microbial biomarkers that could distinguish RM from non-RM to explore whether genes and microbes significantly associated with tumor recurrence and metastasis in LUSC patients were significantly associated with patient survival. Overall survival (OS), progression-free interval (PFI), disease-specific survival (DSS), and disease-free interval (DFI) were chosen as survival data, respectively. The survival analysis regression model was constructed by the ‘coxph’ function of the R package, with biomarker as the model-independent variable and survival time as the model-dependent variable. The risk scores for each LUSC patient were calculated from the survival analysis regression model as follows: risk score*_i_* = β_1_biomarker*_i_*_1_ + β_2_biomarker*_i_*_2_ + … + β*_n_*biomarker*_in_*, where *i* denotes the *i*-th LUSC patient, *n* is the number of biomarkers, and β*_n_* is the regression coefficient corresponding to the *n*-th biomarker in the survival analysis regression model. We plotted the time-dependent ROC curves for OS, PFI, DSS, and DPI separately by using the R package ‘survivalROC’.

We selected the point on the time-dependent ROC curve with the largest difference between true positives and false positives as the cutoff value of the risk score, and patients above this cutoff value were defined as the high-risk group and those below this value as the low-risk group. The survival curve was performed by using the Kaplan-Meier method and the log rank test was used to compare the difference in survival probability with the R package ‘survival’. A *P* value of <0.05 was considered statistically significant.

### Code availability.

All programming scripts to access, manage, and run data on LUAD and LUSC as well as development of the interaction analysis, ML pipelines, survival analysis, and so forth can be found at our GitHub repository link: https://github.com/xfchow/gene_microbiota_interaction. Our pipeline is also publicly shareable and available upon reasonable request of the corresponding author.

### Data availability.

The gene expression, tissue microbiome, and clinical data that support the findings of this study are openly available at https://portal.gdc.cancer.gov and ftp://ftp.microbio.me/pub/cancer_microbiome_analysis/TCGA/.

## References

[1] Ferlay J, Colombet M, Soerjomataram I, Mathers C, Parkin DM, Pineros M, Znaor A, Bray F. 2019. Estimating the global cancer incidence and mortality in 2018: GLOBOCAN sources and methods. Int J Cancer 144:1941–1953. doi:10.1002/ijc.31937. 30350310

[2] Lazarus KA, Hadi F, Zambon E, Bach K, Santolla MF, Watson JK, Correia LL, Das M, Ugur R, Pensa S, Becker L, Campos LS, Ladds G, Liu P, Evan GI, McCaughan FM, Le Quesne J, Lee JH, Calado D, Khaled WT. 2018. BCL11A interacts with SOX2 to control the expression of epigenetic regulators in lung squamous carcinoma. Nat Commun 9:3327. doi:10.1038/s41467-018-05790-5. 30127402 PMC6102279

[3] Travis WD. 2020. Lung cancer pathology. Clin Chest Med 41:67–85. doi:10.1016/j.ccm.2019.11.001. 32008630

[4] Gu C, Wang R, Pan X, Huang Q, Luo J, Zheng J, Wang Y, Shi J, Chen H. 2017. Comprehensive study of prognostic risk factors of patients underwent pneumonectomy. J Cancer 8:2097–2103. doi:10.7150/jca.19454. 28819411 PMC5559972

[5] Chen J, Gu C, Chen X, Dai C, Zhao S, Xie H, Fei K, Chen C. 2021. Clinicopathological and prognostic analyses of 86 resected pulmonary lymphoepithelioma-like carcinomas. J Surg Oncol 123:544–552. doi:10.1002/jso.26276. 33125732

[6] Chen B, Li R, Zhang J, Xu L, Jiang F. 2022. Genomic landscape of metastatic lymph nodes and primary tumors in non-small-cell lung cancer. Pathol Oncol Res 28:1610020. doi:10.3389/pore.2022.1610020. 35783357 PMC9243222

[7] Wang B-Y, Huang J-Y, Chen H-C, Lin C-H, Lin S-H, Hung W-H, Cheng Y-F. 2020. The comparison between adenocarcinoma and squamous cell carcinoma in lung cancer patients. J Cancer Res Clin Oncol 146:43–52. doi:10.1007/s00432-019-03079-8. 31705294 PMC11804334

[8] Yang Y, Wang M, Liu B. 2019. Exploring and comparing of the gene expression and methylation differences between lung adenocarcinoma and squamous cell carcinoma. J Cell Physiol 234:4454–4459. doi:10.1002/jcp.27240. 30317601

[9] Qiu ZW, Bi JH, Gazdar AF, Song K. 2017. Genome-wide copy number variation pattern analysis and a classification signature for non-small cell lung cancer. Genes Chromosomes Cancer 56:559–569. doi:10.1002/gcc.22460. 28379620 PMC5555588

[10] Yang Y, Xu L, Sun L, Zhang P, Farid SS. 2022. Machine learning application in personalised lung cancer recurrence and survivability prediction. Comput Struct Biotechnol J 20:1811–1820. doi:10.1016/j.csbj.2022.03.035. 35521553 PMC9043969

[11] Zhang L, Chen J, Cheng T, Yang H, Li H, Pan C. 2020. Identification of the key genes and characterizations of tumor immune microenvironment in lung adenocarcinoma (LUAD) and lung squamous cell carcinoma (LUSC). J Cancer 11:4965–4979. doi:10.7150/jca.42531. 32742444 PMC7378909

[12] Zhang Y, Chang L, Yang Y, Fang W, Guan Y, Wu A, Hong S, Zhou H, Chen G, Chen X, Zhao S, Zheng Q, Pan H, Zhang L, Long H, Yang H, Wang X, Wen Z, Wang J, Yang H, Xia X, Zhao Y, Hou X, Ma Y, Zhou T, Zhang Z, Zhan J, Huang Y, Zhao H, Zhou N, Yi X, Zhang L. 2019. Intratumor heterogeneity comparison among different subtypes of non-small-cell lung cancer through multi-region tissue and matched ctDNA sequencing. Mol Cancer 18:7. doi:10.1186/s12943-019-0939-9. 30626401 PMC6325778

[13] Cullin N, Azevedo Antunes C, Straussman R, Stein-Thoeringer CK, Elinav E. 2021. Microbiome and cancer. Cancer Cell 39:1317–1341. doi:10.1016/j.ccell.2021.08.006. 34506740

[14] Yang M, Yang H, Ji L, Hu X, Tian G, Wang B, Yang J. 2022. A multi-omics machine learning framework in predicting the survival of colorectal cancer patients. Comput Biol Med 146:105516. doi:10.1016/j.compbiomed.2022.105516. 35468406

[15] Peters BA, Hayes RB, Goparaju C, Reid C, Pass HI, Ahn J. 2019. The microbiome in lung cancer tissue and recurrence-free survival. Cancer Epidemiol Biomarkers Prev 28:731–740. doi:10.1158/1055-9965.EPI-18-0966. 30733306 PMC6449216

[16] Yu T, Guo F, Yu Y, Sun T, Ma D, Han J, Qian Y, Kryczek I, Sun D, Nagarsheth N, Chen Y, Chen H, Hong J, Zou W, Fang JY. 2017. Fusobacterium nucleatum promotes chemoresistance to colorectal cancer by modulating autophagy. Cell 170:548–563.e16. doi:10.1016/j.cell.2017.07.008. 28753429 PMC5767127

[17] Bullman S, Pedamallu CS, Sicinska E, Clancy TE, Zhang X, Cai D, Neuberg D, Huang K, Guevara F, Nelson T. 2017. Analysis of *Fusobacterium* persistence and antibiotic response in colorectal cancer. Science 358:1443–1448. doi:10.1126/science.aal5240. 29170280 PMC5823247

[18] Patnaik SK, Cortes EG, Kannisto ED, Punnanitinont A, Dhillon SS, Liu S, Yendamuri S. 2021. Lower airway bacterial microbiome may influence recurrence after resection of early-stage non-small cell lung cancer. J Thorac Cardiovasc Surg 161:419–429.e16. doi:10.1016/j.jtcvs.2020.01.104. 32340803

[19] Tang WF, Wu M, Bao H, Xu Y, Lin JS, Liang Y, Zhang Y, Chu XP, Qiu ZB, Su J, Zhang JT, Zhang C, Xu FP, Chen JH, Fu R, Chen Y, Yang T, Chen QK, Wu TT, Wu X, Shao Y, Zheng JT, Xie Z, Lv ZY, Dong S, Wu YL, Zhong WZ. 2021. Timing and origins of local and distant metastases in lung cancer. J Thorac Oncol 16:1136–1148. doi:10.1016/j.jtho.2021.02.023. 33722707

[20] Hung JJ, Jeng WJ, Hsu WH, Chou TY, Huang BS, Wu YC. 2012. Predictors of death, local recurrence, and distant metastasis in completely resected pathological stage-I non-small-cell lung cancer. J Thorac Oncol 7:1115–1123. doi:10.1097/JTO.0b013e31824cbad8. 22592210

[21] Li L, Liu YD, Zhan YT, Zhu YH, Li Y, Xie D, Guan XY. 2018. High levels of CCL2 or CCL4 in the tumor microenvironment predict unfavorable survival in lung adenocarcinoma. Thorac Cancer 9:775–784. doi:10.1111/1759-7714.12643. 29722145 PMC6026602

[22] Kaishang Z, Xue P, Shaozhong Z, Yingying F, Yan Z, Chanjun S, Zhenzhen L, Xiangnan L. 2018. Elevated expression of Twinfilin-1 is correlated with inferior prognosis of lung adenocarcinoma. Life Sci 215:159–169. doi:10.1016/j.lfs.2018.10.067. 30391462

[23] Jin C, Lagoudas GK, Zhao C, Bullman S, Bhutkar A, Hu B, Ameh S, Sandel D, Liang XS, Mazzilli S, Whary MT, Meyerson M, Germain R, Blainey PC, Fox JG, Jacks T. 2019. Commensal microbiota promote lung cancer development via γδ T cells. Cell 176:998–1013.e16. doi:10.1016/j.cell.2018.12.040. 30712876 PMC6691977

[24] Bao Z, Lu G, Cui D, Yao Y, Yang G, Zhou J. 2016. IL-17A-producing T cells are associated with the progression of lung adenocarcinoma. Oncol Rep 36:641–650. doi:10.3892/or.2016.4837. 27277161 PMC4933549

[25] Dayama G, Priya S, Niccum DE, Khoruts A, Blekhman R. 2020. Interactions between the gut microbiome and host gene regulation in cystic fibrosis. Genome Med 12:12. doi:10.1186/s13073-020-0710-2. 31992345 PMC6988342

[26] Greathouse KL, White JR, Vargas AJ, Bliskovsky VV, Beck JA, von Muhlinen N, Polley EC, Bowman ED, Khan MA, Robles AI, Cooks T, Ryan BM, Padgett N, Dzutsev AH, Trinchieri G, Pineda MA, Bilke S, Meltzer PS, Hokenstad AN, Stickrod TM, Walther-Antonio MR, Earl JP, Mell JC, Krol JE, Balashov SV, Bhat AS, Ehrlich GD, Valm A, Deming C, Conlan S, Oh J, Segre JA, Harris CC. 2018. Interaction between the microbiome and TP53 in human lung cancer. Genome Biol 19:123. doi:10.1186/s13059-018-1501-6. 30143034 PMC6109311

[27] Pijnenborg JM, van de Broek L, Dam de Veen GC, Roemen GM, de Haan J, van Engeland M, Voncken JW, Groothuis PG. 2006. TP53 overexpression in recurrent endometrial carcinoma. Gynecol Oncol 100:397–404. doi:10.1016/j.ygyno.2005.09.056. 16271749

[28] Hassan NM, Tada M, Hamada J, Kashiwazaki H, Kameyama T, Akhter R, Yamazaki Y, Yano M, Inoue N, Moriuchi T. 2008. Presence of dominant negative mutation of TP53 is a risk of early recurrence in oral cancer. Cancer Lett 270:108–119. doi:10.1016/j.canlet.2008.04.052. 18555592

[29] Shi M, Wang J, Zhang C. 2020. Integration of cancer genomics data for tree-based dimensionality reduction and cancer outcome prediction. Mol Inform 39:e1900028. doi:10.1002/minf.201900028. 31490641

[30] Poore GD, Kopylova E, Zhu Q, Carpenter C, Fraraccio S, Wandro S, Kosciolek T, Janssen S, Metcalf J, Song SJ, Kanbar J, Miller-Montgomery S, Heaton R, McKay R, Patel SP, Swafford AD, Knight R. 2020. Microbiome analyses of blood and tissues suggest cancer diagnostic approach. Nature 579:567–574. doi:10.1038/s41586-020-2095-1. 32214244 PMC7500457

[31] Oyama T, Sugio K, Uramoto H, Onizuka T, Iwata T, Nozoe T, Takenoyama M, Hanagiri T, Isse T, Kawamoto T, Yasumoto K. 2007. P2-049: cytochrome P450 expression in non-small cell lung cancer. J Thorac Oncol 2:S509–S510. doi:10.1097/01.JTO.0000283513.59291.2f.

[32] Oyama T, Uramoto H, Kagawa N, Yoshimatsu T, Osaki T, Nakanishi R, Nagaya H, Kaneko K, Muto M, Kawamoto T, Tanaka F, Gotoh A. 2012. Cytochrome P450 in non-small cell lung cancer related to exogenous chemical metabolism. Front Biosci (Schol Ed) 4:1539–1546. doi:10.2741/s350. 22652890

[33] Zhen Y, Wu Y, Wu Y. 2007. Relation between cytochrome P450 2D6 and lung cancer susceptibility caused by smoking. Wei Sheng Yan Jiu 36:112–116. (In Chinese.) 17424863

[34] Winton T, Livingston R, Johnson D, Rigas J, Johnston M, Butts C, Cormier Y, Goss G, Inculet R, Vallieres E, Fry W, Bethune D, Ayoub J, Ding K, Seymour L, Graham B, Tsao M-S, Gandara D, Kesler K, Demmy T, Shepherd F, National Cancer Institute of Canada Clinical Trials Group, National Cancer Institute of the United States Intergroup JBR.10 Trial Investigators. 2005. Vinorelbine plus cisplatin vs. observation in resected non-small-cell lung cancer. N Engl J Med 352:2589–2597. doi:10.1056/NEJMoa043623. 15972865

[35] Subotic D, Van Schil P, Grigoriu B. 2016. Optimising treatment for post-operative lung cancer recurrence. Eur Respir J 47:374–378. doi:10.1183/13993003.01490-2015. 26828046

[36] Hung JJ, Hsu WH, Hsieh CC, Huang BS, Huang MH, Liu JS, Wu YC. 2009. Post-recurrence survival in completely resected stage I non-small cell lung cancer with local recurrence. Thorax 64:192–196. doi:10.1136/thx.2007.094912. 19252018

[37] Liu H, Qiu C, Wang B, Bing P, Tian G, Zhang X, Ma J, He B, Yang J. 2021. Evaluating DNA methylation, gene expression, somatic mutation, and their combinations in inferring tumor tissue-of-origin. Front Cell Dev Biol 9:619330. doi:10.3389/fcell.2021.619330. 34012960 PMC8126648

[38] Anttila S, Raunio H, Hakkola J. 2011. Cytochrome P450-mediated pulmonary metabolism of carcinogens: regulation and cross-talk in lung carcinogenesis. Am J Respir Cell Mol Biol 44:583–590. doi:10.1165/rcmb.2010-0189RT. 21097654

[39] Sanderson JT. 2006. The steroid hormone biosynthesis pathway as a target for endocrine-disrupting chemicals. Toxicol Sci 94:3–21. doi:10.1093/toxsci/kfl051. 16807284

[40] Siegfried JM, Stabile LP. 2014. Estrogenic steroid hormones in lung cancer. Semin Oncol 41:5–16. doi:10.1053/j.seminoncol.2013.12.009. 24565577 PMC4001725

[41] Nabi H, Provencher L, Diorio C. 2018. RE: smoking, sex, and non-small cell lung cancer: steroid hormone receptors in tumor tissue (S0424). J Natl Cancer Inst 110:1422–1423. doi:10.1093/jnci/djy068. 29688493

[42] Zhao Y, Liu Y, Li S, Peng Z, Liu X, Chen J, Zheng X. 2021. Role of lung and gut microbiota on lung cancer pathogenesis. J Cancer Res Clin Oncol 147:2177–2186. doi:10.1007/s00432-021-03644-0. 34018055 PMC8236441

[43] Mao Q, Ma W, Wang Z, Liang Y, Zhang T, Yang Y, Xia W, Jiang F, Hu J, Xu L. 2020. Differential flora in the microenvironment of lung tumor and paired adjacent normal tissues. Carcinogenesis 41:1094–1103. doi:10.1093/carcin/bgaa044. 32658980

[44] Fan Z, Zhang Q, Feng L, Wang L, Zhou X, Han J, Li D, Liu J, Zhang X, Zuo J, Zou X, Cai Y, Sun Y, Wang Y. 2022. Genomic landscape and prognosis of patients with TP53-mutated non-small cell lung cancer. Ann Transl Med 10:188. doi:10.21037/atm-22-412. 35280362 PMC8908146

[45] Xu F, Lin H, He P, He L, Chen J, Lin L, Chen Y. 2020. A TP53-associated gene signature for prediction of prognosis and therapeutic responses in lung squamous cell carcinoma. Oncoimmunology 9:1731943. doi:10.1080/2162402X.2020.1731943. 32158625 PMC7051188

[46] Li DD, Fu ZQ, Lin Q, Zhou Y, Zhou QB, Li ZH, Tan LP, Chen RF, Liu YM. 2015. Linc00675 is a novel marker of short survival and recurrence in patients with pancreatic ductal adenocarcinoma. World J Gastroenterol 21:9348–9357. doi:10.3748/wjg.v21.i31.9348. 26309360 PMC4541386

[47] Zhuo H, Tang J, Lin Z, Jiang R, Zhang X, Ji J, Wang P, Sun B. 2016. The aberrant expression of MEG3 regulated by UHRF1 predicts the prognosis of hepatocellular carcinoma. Mol Carcinog 55:209–219. doi:10.1002/mc.22270. 25641194

[48] Yan J, Guo X, Xia J, Shan T, Gu C, Liang Z, Zhao W, Jin S. 2014. MiR-148a regulates MEG3 in gastric cancer by targeting DNA methyltransferase 1. Med Oncol 31:879. doi:10.1007/s12032-014-0879-6. 24515776

[49] Lu KH, Li W, Liu XH, Sun M, Zhang ML, Wu WQ, Xie WP, Hou YY. 2013. Long non-coding RNA MEG3 inhibits NSCLC cells proliferation and induces apoptosis by affecting p53 expression. BMC Cancer 13:461. doi:10.1186/1471-2407-13-461. 24098911 PMC3851462

[50] Krishnamurthy N, Kurzrock R. 2018. Targeting the Wnt/beta-catenin pathway in cancer: update on effectors and inhibitors. Cancer Treat Rev 62:50–60. doi:10.1016/j.ctrv.2017.11.002. 29169144 PMC5745276

[51] Clevers H. 2006. Wnt/beta-catenin signaling in development and disease. Cell 127:469–480. doi:10.1016/j.cell.2006.10.018. 17081971

[52] Shan Z, An N, Qin J, Yang J, Sun H, Yang W. 2018. Long non-coding RNA Linc00675 suppresses cell proliferation and metastasis in colorectal cancer via acting on miR-942 and Wnt/beta-catenin signaling. Biomed Pharmacother 101:769–776. doi:10.1016/j.biopha.2018.02.123. 29524886

[53] Ge C, Wu S, Wang W, Liu Z, Zhang J, Wang Z, Li R, Zhang Z, Li Z, Dong S, Wang Y, Xue Y, Yang J, Tan Q, Wang Z, Song X. 2015. miR-942 promotes cancer stem cell-like traits in esophageal squamous cell carcinoma through activation of Wnt/β-catenin signalling pathway. Oncotarget 6:10964–10977. doi:10.18632/oncotarget.3696. 25844602 PMC4484432

[54] Tao L, Xue D, Shen D, Ma W, Zhang J, Wang X, Zhang W, Wu L, Pan K, Yang Y, Nwosu ZC, Dooley S, Seki E, Liu C. 2018. MicroRNA-942 mediates hepatic stellate cell activation by regulating BAMBI expression in human liver fibrosis. Arch Toxicol 92:2935–2946. doi:10.1007/s00204-018-2278-9. 30097701 PMC6590087

[55] Wang Y, Wu Y, Xie S. 2022. CircPTK2 inhibits cell cisplatin (CDDP) resistance by targeting miR-942/TRIM16 axis in non-small cell lung cancer (NSCLC). Bioengineered 13:3651–3664. doi:10.1080/21655979.2021.2024321. 35230201 PMC8973636

[56] Wei K, Ma Z, Yang F, Zhao X, Jiang W, Pan C, Li Z, Pan X, He Z, Xu J, Wu W, Xia Y, Chen L. 2022. M2 macrophage-derived exosomes promote lung adenocarcinoma progression by delivering miR-942. Cancer Lett 526:205–216. doi:10.1016/j.canlet.2021.10.045. 34838826

[57] Bagnoli M, De Cecco L, Granata A, Nicoletti R, Marchesi E, Alberti P, Valeri B, Libra M, Barbareschi M, Raspagliesi F, Mezzanzanica D, Canevari S. 2011. Identification of a chrXq27.3 microRNA cluster associated with early relapse in advanced stage ovarian cancer patients. Oncotarget 2:1265–1278. doi:10.18632/oncotarget.401. 22246208 PMC3282083

[58] Fan S, Wang L. 2021. N(6)-methyladenosine-regulated LINC00675 suppress the proliferation, migration and invasion of breast cancer cells via inhibiting miR-513b-5p. Bioengineered 12:10690–10702. doi:10.1080/21655979.2021.2001905. 34738869 PMC8810037

[59] O’Byrne KJ, Koukourakis MI, Giatromanolaki A, Cox G, Turley H, Steward WP, Gatter K, Harris AL. 2000. Vascular endothelial growth factor, platelet-derived endothelial cell growth factor and angiogenesis in non-small-cell lung cancer. Br J Cancer 82:1427–1432. doi:10.1054/bjoc.1999.1129. 10780522 PMC2363365

[60] Kuo MW, Wang CH, Wu HC, Chang SJ, Chuang YJ. 2011. Soluble THSD7A is an N-glycoprotein that promotes endothelial cell migration and tube formation in angiogenesis. PLoS One 6:e29000. doi:10.1371/journal.pone.0029000. 22194972 PMC3237571

[61] Carmeliet P, Jain RK. 2000. Angiogenesis in cancer and other diseases. Nature 407:249–257. doi:10.1038/35025220. 11001068

[62] D’Alessandro-Gabazza CN, Mendez-Garcia C, Hataji O, Westergaard S, Watanabe F, Yasuma T, Toda M, Fujimoto H, Nishihama K, Fujiwara K, Taguchi O, Kobayashi T, Mackie RI, Cann I, Gabazza EC. 2018. Identification of halophilic microbes in lung fibrotic tissue by oligotyping. Front Microbiol 9:1892. doi:10.3389/fmicb.2018.01892. 30233503 PMC6127444

[63] Flemer B, Lynch DB, Brown JM, Jeffery IB, Ryan FJ, Claesson MJ, O’Riordain M, Shanahan F, O’Toole PW. 2017. Tumour-associated and non-tumour-associated microbiota in colorectal cancer. Gut 66:633–643. doi:10.1136/gutjnl-2015-309595. 26992426 PMC5529966

[64] Najafi S, Abedini F, Azimzadeh Jamalkandi S, Shariati P, Ahmadi A, Gholami Fesharaki M. 2021. The composition of lung microbiome in lung cancer: a systematic review and meta-analysis. BMC Microbiol 21:315. doi:10.1186/s12866-021-02375-z. 34763672 PMC8582175

[65] Salachan PV, Sorensen KD. 2022. Dysbiotic microbes and how to find them: a review of microbiome profiling in prostate cancer. J Exp Clin Cancer Res 41:31. doi:10.1186/s13046-021-02196-y. 35065652 PMC8783429

[66] Oksanen J, Blanchet FG, Kindt R, Legendre P, Minchin P, O’Hara B, Simpson G, Solymos P, Stevens H, Wagner H. 2015. Vegan: community ecology package. R package version 22-1 2:1-2.

[67] Costa-Silva J, Domingues D, Lopes FM. 2017. RNA-Seq differential expression analysis: an extended review and a software tool. PLoS One 12:e0190152. doi:10.1371/journal.pone.0190152. 29267363 PMC5739479

[68] Fazlollahi M, Lee TD, Andrade J, Oguntuyo K, Chun Y, Grishina G, Grishin A, Bunyavanich S. 2018. The nasal microbiome in asthma. J Allergy Clin Immunol 142:834–843.e2. doi:10.1016/j.jaci.2018.02.020. 29518419 PMC6123291

[69] Davies JC. 2002. Pseudomonas aeruginosa in cystic fibrosis: pathogenesis and persistence. Paediatr Respir Rev 3:128–134. doi:10.1016/S1526-0550(02)00003-3. 12297059

