# Supplementary figures and images for: Intratumoral Microbiota-Host Interactions Shape the Variability of Lung Adenocarcinoma and Lung Squamous Cell Carcinoma in Recurrence and Metastasis
Source: Microbiol Spectr. 2023 Apr 19;11(3):e03738-22. doi: 10.1128/spectrum.03738-22 (PMC10269859; doi:10.1128/spectrum.03738-22)

**A**

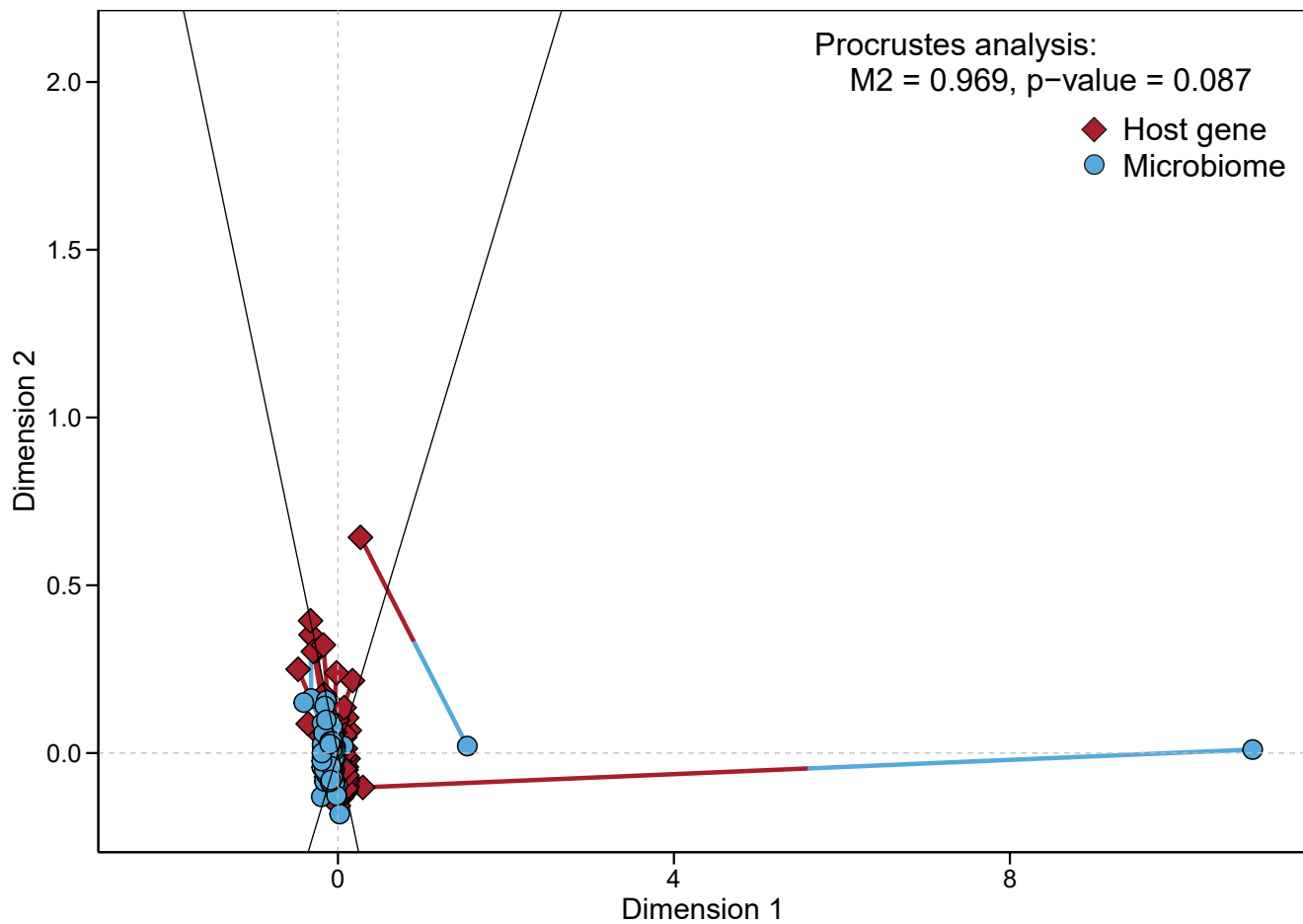

**B**

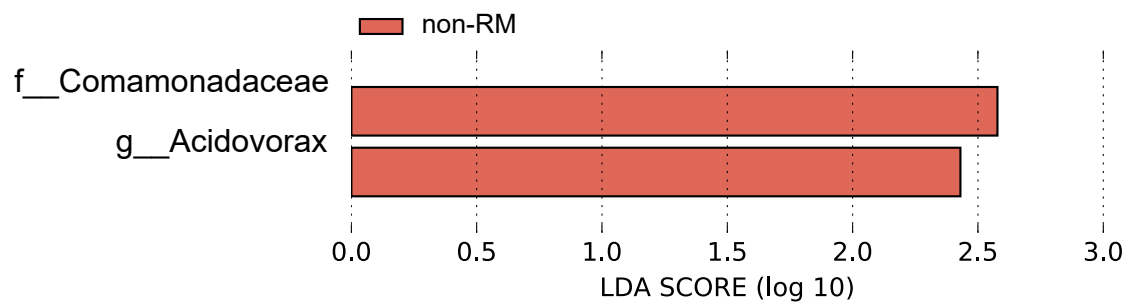

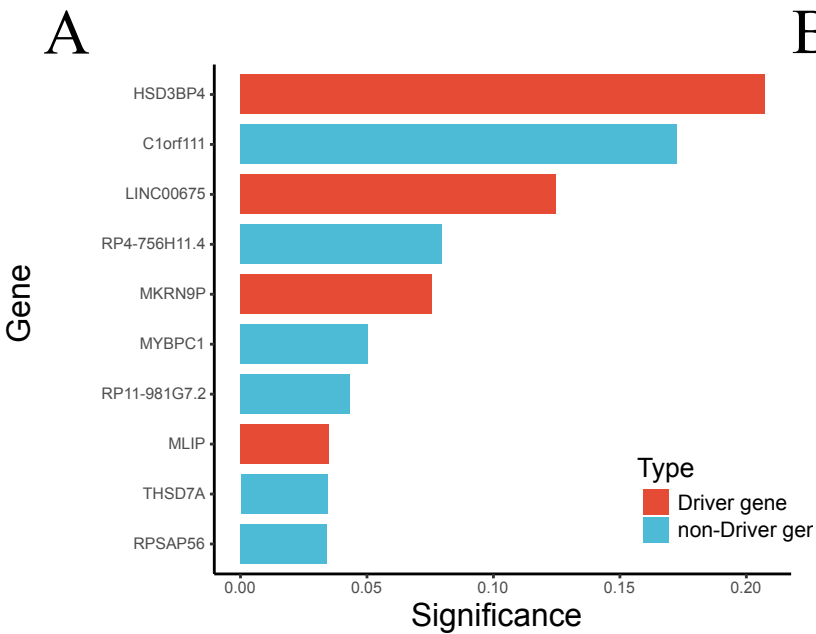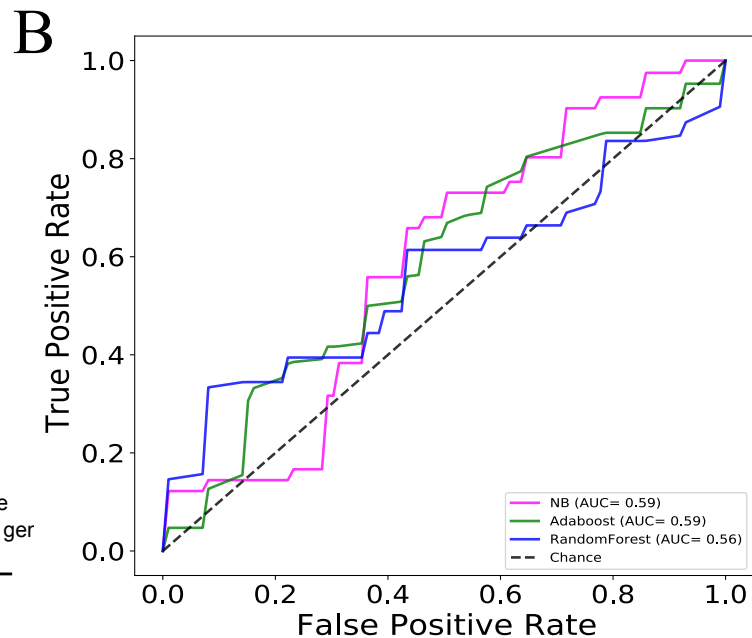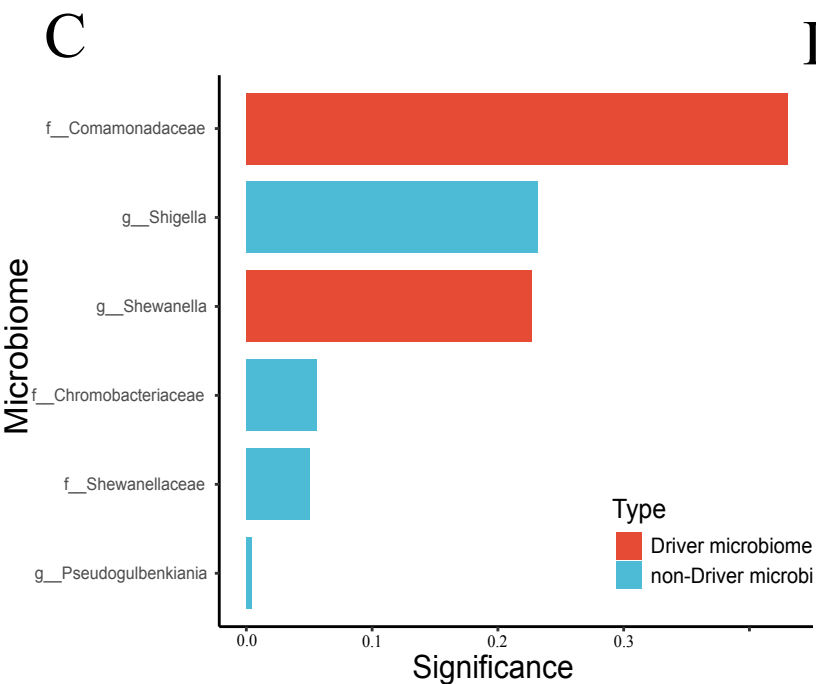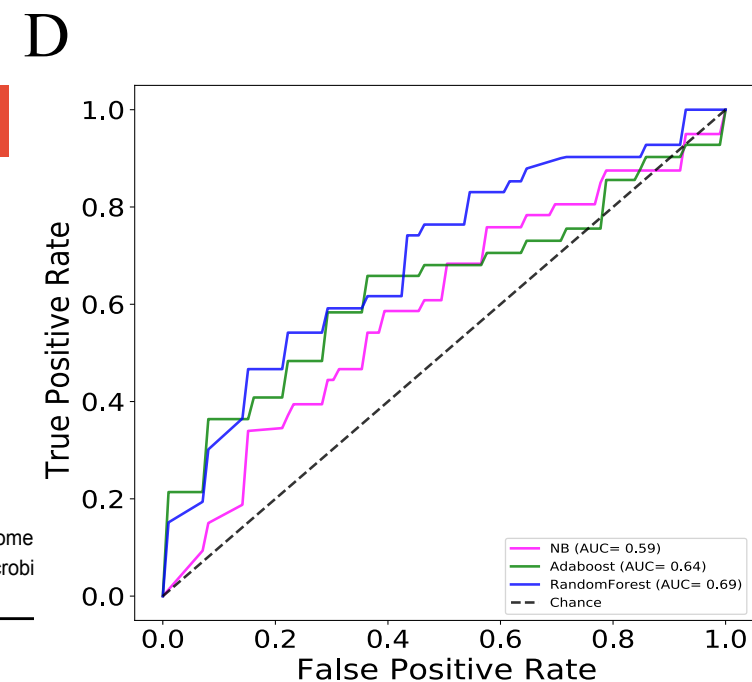

Supplement: Supplemental file 1 — Supplemental material. Download spectrum.03738-22-s0001.pdf, PDF file, 0.2 MB [file spectrum.03738-22-s0001.pdf]
